# Disparities in fruit and vegetable intake at the intersection of gender and education in northern Sweden: a cross-sectional study

**DOI:** 10.1186/s40795-022-00641-5

**Published:** 2022-12-12

**Authors:** Thomas Vogt, Per E. Gustafsson

**Affiliations:** Department of Epidemiology and Global Health, Umeå University, Umeå, Sweden

**Keywords:** Intersectionality, Joint disparity, Referent disparities, Excess intersectional disparity, Discriminatory accuracy, Fruits, Vegetables, Gender, Education, Sweden

## Abstract

**Background:**

Even though the existence of inequalities in fruit and vegetable consumption has been well established, it is not clear how it is patterned across intersections of multiple social positions and identities. This study aims to investigate disparities in fruit and vegetable intake between groups at the intersection of education and gender in northern Sweden, and to estimate the discriminatory accuracy of the intersectional groups.

**Methods:**

Cross-sectional data from the 2018 Health on Equal Terms survey conducted in four regions in northern Sweden was used (*N* = 21,853). Four intersectional groups were created: high and low educated men, and high and low educated women. Prevalence differences corresponding to joint, referent, and excess intersectional inequalities, were estimated for three outcomes: inadequate fruit and vegetable intake combined, inadequate fruit intake, and inadequate vegetable intake. The discriminatory accuracy of the intersectional groups was estimated by the area under the receiver operating characteristic curve.

**Results:**

Low educated men had the highest prevalence of inadequate intake of fruits and vegetables combined (81.4%), fruits (83.4%), and vegetables (84.9%), while high educated women had the lowest (47.7, 60.2, and 51.8%, respectively). The joint disparities between high educated women and low educated men were both significant and substantial for all outcomes (34.6 percentage points (pp.), 25.2 pp., and 31.2 pp., adjusted), although differences in magnitude were noted between fruit and vegetable intake. The joint disparities were mostly explained by the two referent disparities for gender and education. The excess intersectional disparity - the part of the joint disparity not explained by either referent disparity – was negative for all three outcomes (-5.5 pp., − 4.2 pp., and − 4.6 pp. respectively, adjusted). The discriminatory accuracy of the intersectional groups was moderate (0.67, 0.65, and 0.68 respectively).

**Conclusions:**

An intersectional approach can provide a more detailed view of inequalities in fruit and vegetable consumption between groups combining several social positions. The moderate discriminatory accuracy observed here suggests that interventions and policies aiming to reduce diet inequalities should not solely be targeted at certain groups, but also be universal.

## Background

A suboptimal diet could be the risk factor with the greatest impact on mortality worldwide, with low fruit and low vegetable intake among the main contributors [1], associated with increased risk of coronary heart disease, stroke, total cardiovascular disease, total cancer, and all-cause mortality [2]. An adequate fruit and vegetable intake is therefore considered critical for a healthy diet in recommendations and guidelines worldwide [3–5], and for a healthy lifestyle together with other factors such as physical activity [4].

The burden linked to inadequate consumption of fruits and vegetables is however not shared equally, with disparities in consumption demonstrated both between and within countries [1, 6, 7]. Within-country differences in fruit and vegetable intake in high-income countries have been shown for socioeconomic status [6–10], gender [6, 9], as well as demographic characteristics such as marital status [7], age [9, 10], and region of residence [7]. Inequitable patterns in nutrition may therefore be shaped differently in different contexts, depending on the specific configuration of those characteristics. Because of their strong impact on health outcomes, disparities in fruit and vegetable intake can also represent one of the pathways by which inequalities in health are produced and maintained in the population [11], and therefore one possible target for the promotion of equity in health.

Higher consumption of fruits and vegetables among socioeconomically advantaged groups [6–10, 12, 13] has been attributed to better knowledge about the health benefits of eating fruits and vegetables, a greater sense of control over one’s health, and could also be a way for each group to distinguish itself from the other by adhering to a different pattern of fruit and vegetable intake [14]. The pattern is different for gender, where women tend to engage in health-promoting behaviors to a greater degree than men [15–18], including a higher intake of fruits and vegetables [6, 19, 20]. A common explanation for the gender inequality favoring the structurally disadvantaged group of women is that men display more risk behavior as a way to construct their masculinity and assert their power over women and other men [17]. Food preparation skills also play a role in dietary intake [21] and these skills may be distributed differently by gender. However, men in Sweden might consider the acquisition of cooking skills as desirable and a responsibility [22].

Although fruit and vegetable consumption tends to be socially patterned in conflicting manners across education on the one hand and between genders on the other, there is little empirical research on how fruit and vegetable consumption is patterned across combined social positions. Such complex inequalities in health and health behaviors are however the focus of intersectionality theory, which is an analytical framework specifically considering the intersection of several social identities and positions [23]. This theory was first introduced by law scholar Kimberlé Crenshaw in 1989 [24]. Over the following decades the theory spread ubiquitously and was further developed across mainly qualitatively oriented social sciences, which has resulted in a considerable heterogeneity in the specific interpretation and application of intersectionality [25]. The last few years have seen a concentrated effort to translate and integrate intersectionality into social epidemiology and population health research [23, 26–28], with a potential to give more precision to public health research and provide a more detailed and nuanced picture of the way social inequalities contribute to inequalities in health [26, 29].

Much of the discussion on integrating intersectionality into social epidemiology [27, 28, 30–32] has centered on the analytical operationalization of three hypothesized patterns of outcomes in disadvantaged intersectional groups: i) the outcome is patterned by only one of the group’s multiple positions; ii) the combination of two positions leads to a double disadvantage and has therefore an additive effect, and iii) the disadvantage experienced is a product of the specific intersection, and is larger or smaller than the sum of the disadvantage associated with the positions characterizing the intersectional group [24]. Various main approaches of applications have been identified in epidemiology [31]. In the first, intersections between groups are represented by cross-classified categories, on which an outcome will be regressed, conventionally using multiplicative-scale models and thus estimating relative inequalities. In the second and more recent approach described by Jackson et al. [33], a joint disparity in the outcome is estimated on the additive scale between the group who is doubly disadvantaged and the one who is doubly advantaged, which is subsequently decomposed into the portions attributable to each social position (the referent disparities), and the part attributable to the intersection of the two disadvantages (the excess intersectional disparity). This decomposition has the benefit of resembling the possibilities for an intersectional group to experience disadvantage according to the three hypothesized patterns described above, and has been used to study inequalities in mental health in the US [33] and in Sweden [34], health care utilization in Sweden [35], as well as health behaviors such as smoking [36]. Another recent intersectional analytical approach has emerged within epidemiology in recent years, labelled (Multilevel) Analysis of Individual Heterogeneity and Discriminatory Accuracy ((M)AIHDA) [27, 37, 38], which estimates the degree to which intersectional categories can discriminate effectively between cases and non-cases [27]. These methods specifically provide evidence that is informative when it comes to the principle of proportionate universalism, i.e. universal interventions with a proportionate increased intensity in groups of higher risk, and have been applied in numerous studies on intersectional inequalities, for example concerning self-rated general and mental health [34, 39], ischemic heart disease [37], antidepressant and antibiotic use [40, 41], as well as health behaviors in the form of smoking [38]. Overall, most results have pointed towards a poor to moderate ability of intersectional groups to discriminate between cases and non-cases, which suggests that universal interventions may be more suitable than targeting high-risk groups.

To the authors’ knowledge, no studies have looked at intersectional inequalities in fruit and vegetable intake by gender and education, either in Sweden or elsewhere. This study therefore aims to estimate disparities in fruit and vegetable intake between groups at the intersection of gender and education in northern Sweden. An additional aim is to assess the extent to which the intersectional groups discriminate between adequate and inadequate intake of fruits and vegetables.

## Methods

### Population and design

The data came from the cross-sectional Health on Equal Terms survey. This survey is carried out nationally every (2004–2016) to every second (2016-present) year. Every 4 years, four regions from northern Sweden provide a larger sample to expand the survey. This study uses data from the most recent expanded survey conducted in 2018 in the regions of Norrbotten, Västerbotten, Jämtland/Härjedalen, and Västernorrland.

The 2018 survey consisted of a questionnaire with 64 questions on varied subjects such as mental health, diet, occupation, or healthcare use [42]. Both an online and print version of the questionnaire were offered. The survey results were linked to register data by Statistics Sweden for each individual participant using the unique Swedish personal identity number. Register data included, among other variables, age, sex, education, civil status and region of residence [43].

The target population from which the sample was drawn comprised everyone aged 16 to 84 from Norrbotten, Västerbotten, Jämtland/Härjedalen, and Västernorrland, identified through the Register of the Total Population on November 30th, 2017 [42]. Each region conducted its own sampling procedures in collaboration with the Public Health Agency of Sweden and Statistics Sweden. At the national level, the sample was determined through unrestricted random sampling, and drawn from a target population of *N* = 7,930,792 [42]. At the regional level, random cluster sampling was used, stratified by age and municipality (and in Västerbotten, also by gender). The total target population in the four regions was 711,303 [44–47]. Of the total sample from the four northern regions, *N* = 3510 (6.5% of the sample) were included in the national survey, to which an additional sample of *N* = 50,600 (93.5% of the sample) was selected as part of the expanded survey. *N* = 208 were excluded because they no longer belonged to the population, resulting in a final sample of 53,902 in total. The expanded 2018 survey from the four regions had 23,487 responses in total and a response rate of 43.6% [44–47].

In the present study, only those aged over 20 years were included in order to capture post-secondary school leavers. As a result, 1232 responses were intentionally excluded. Respondents not coded as residing in one of the four relevant regions were also excluded (*n* = 46). And after excluding missing observations (*n* = 356), 21,853 observations remained.

### Variables

#### Fruit and vegetable consumption

Two questionnaire items on frequency of fruit and vegetable intake were used: “how often do you eat green vegetables and root vegetables?” and “how often do you eat fruits and berries?”. Possible responses were on a Likert-like ordinal scale with seven options: “less than once a week or never”, “1–2 times a week”, “3–4 times a week”, “5–6 times a week”, “1 time a day”, “2 times a day”, or “3 times a day or more” [43]. The responses were dichotomized into adequate (0; twice a day or more) versus inadequate (1; less than twice a day) consumption of fruit and vegetables.

In addition, weights were attributed to each level of consumption and a summary variable of combined fruit and vegetable consumption was created, in accordance with the procedures used by the Public Health Agency of Sweden [43]. A combined weight of 0.14 is the lowest possible combined consumption for one person, whereas 6 is the highest [43]. The summary index was divided according to recommendations into adequate (0; at least three servings of fruits and/or vegetables daily) and inadequate (1; less than three servings daily) combined consumption [43].

#### Intersectional groups by gender and education

Gender and education were combined to form the four intersectional positions, both retrieved from population registers. Gender was a binary variable with man and woman as the two possible options. Education was measured as the highest level of formal education attained and then dichotomized into high education and low education. High education was defined as having completed at least one term of tertiary education, while low education was defined as having completed secondary education or less. We adapted this dichotomization from previous work on dietary patterns in Sweden [9, 10].

By cross classifying the binary variables gender and education, four intersectional categories were created: men with high education, men with low education, women with high education, and women with low education. Each group combined two potential social advantages and/or disadvantages. Here, *high education* and *men* were understood to be positions of relative social advantage whereas *low education* and *women* were assumed to be positions of social disadvantage [24, 48]. However, as women tend to display more favorable behavioral outcomes including diet [15–18], for the purpose of this study, high-educated women were considered the doubly advantaged group and low-educated men the doubly disadvantaged group.

#### Covariates: age, civil status, region of residence

Additional covariates were included in the analyses: age was categorized in brackets (21–30, 31–44, 45–64, 65–84); civil status was divided into: married/cohabiting and unmarried/not cohabiting; and region of residence comprised Norrbotten, Västerbotten, Jämtland/Härjedalen, and Västernorrland.

### Statistical analysis

The characteristics for each intersectional group are displayed in Table 1.

**Table 1:** Descriptive statistics of the main variables by intersectional group (*N* = 21,853)^a^

Variable		Four groups at the intersection of gender and education			
	Total, *n* = 21,853 (100.0%)	High educated women, *n* = 4908 (22.5%)	Low educated women, *n* = 6849 (31.3%)	High educated men, *n* = 3147 (14.4%)	Low educated men, *n* = 6949 (31.8%)
Combined fruit and vegetable					
Adequate intake (≥3)	7316 (33.5)	2568 (52.3)	2545 (37.2)	911 (28.9)	1292 (18.6)
Inadequate intake (< 3)	14,537 (66.5)	2340 (47.7)	4304 (62.8)	2236 (71.1)	5657 (81.4)
Fruit					
Adequate intake (≥2)	5981 (27.4)	1954 (39.8)	2189 (32.0)	683 (21.7)	1155 (16.6)
Inadequate intake (< 2)	15,872 (72.6)	2954 (60.2)	4660 (68.0)	2464 (78.3)	5794 (83.4)
Vegetable					
Adequate intake (≥2)	6401 (29.3)	2365 (48.2)	2114 (30.9)	873 (27.7)	1049 (15.1)
Inadequate intake (< 2)	15,452 (70.7)	2543 (51.8)	4735 (69.1)	2274 (72.3)	5900 (84.9)
Age					
21–30 years old	2187 (10.0)	556 (11.3)	675 (9.9)	294 (9.3)	662 (9.5)
31–44 years old	3821 (17.5)	1419 (28.9)	795 (11.6)	709 (22.5)	898 (12.9)
45–64 years old	6988 (32.0)	1668 (34.0)	2119 (30.9)	1053 (33.5)	2148 (30.9)
65–84 years old	8857 (40.5)	1265 (25.8)	3260 (47.6)	1091 (34.7)	3241 (46.6)
Civil status					
Married/cohabiting	10,954 (50.1)	2483 (50.6)	3275 (47.8)	1725 (54.8)	3471 (49.9)
Unmarried/not cohabiting	10,899 (49.9)	2425 (49.4)	3574 (52.2)	1422 (45.2)	3478 (50.1)
Region					
Västernorrland	4632 (21.2)	1039 (21.2)	1395 (20.4)	737 (23.4)	1461 (21.0)
Jämtland/Härjedalen	4266 (19.5)	996 (20.3)	1310 (19.1)	575 (18.3)	1385 (19.9)
Västerbotten	4409 (20.2)	940 (19.2)	1477 (21.6)	528 (16.8)	1464 (21.1)
Norrbotten	8546 (39.1)	1933 (39.4)	2667 (38.9)	1307 (41.5)	2639 (38.0)

^a^ Numbers shown are N(%). Percentages are column percentages (except for the header row)

To study the disparities in fruit and vegetable intake between the four intersectional positions, a method previously described by Jackson, et al. [33] was used, estimating four disparities with the doubly advantaged position as the reference group. The joint disparity is the prevalence difference of the outcome (inadequate intake of fruits and vegetables) between the intersectional groups of double disadvantage (low educated men) and double advantage (high educated women). The joint disparity can be further decomposed into referent and excess disparities. The referent disparities are differences in prevalence between one middle group and the reference group of double advantage. In this study, the referent disparity for education equals the prevalence difference in fruit and vegetable intake between low educated women and high educated women, and the referent disparity for gender, the corresponding prevalence difference between high educated men and high educated women. The fourth disparity is the excess intersectional disparity, which is the (positive or negative) difference in prevalence that remains after both referent disparities have been subtracted to the joint disparity and is equivalent to an additive-scale interaction term. That is, the joint disparity equals the sum of the two referent disparities and the excess intersectional disparity:

$$\begin{array}{c}\text{Joint}\;\text{disparity}=\text{Referent}\;\text{disparity}(1)+\text{Referent}\;\text{disparity}(2)\\ \kern2em+\text{Excess}\;\text{intersectional}\;\text{disparity}\end{array}$$

Stata 16.1 was used to carry out the analysis. A generalized linear model with a binomial family distribution specified for the outcome, and an identity link function [49, 50] was used to estimate the joint and referent disparities, while post-estimation of linear combinations of parameters were used to determine the excess intersectional disparity. The generalized linear models were run on the cross-classified intersectional variable separately for each of the three outcome variables, in unadjusted analyses and analyses adjusting for covariates.

Each disparity is expressed as prevalence differences, in percentage points (pp.) with 95% confidence interval. The referent disparities and the excess intersectional disparity were also descriptively expressed as percentages of the joint disparity. A positive estimate for the joint or referent disparity would indicate a higher prevalence of inadequate intake among low educated men, high educated men or low educated women compared to the reference group of high educated women. A zero estimate for the excess intersectional disparity means that the joint disparity is equal to the sum of the two referent disparities, equivalent to an additive effect of gender and education on the outcome. In contrast, a positive (or negative) estimate of excess intersectional disparity would indicate that the prevalence among low educated men was greater (or lower) than what is to be expected from simply considering the two disadvantages as independent and additive to each other.

Discriminatory accuracy was estimated by calculating the Area Under the Receiver Operating Characteristic curve (AU-ROC), using a logistic regression model [51]. This yield results ranging from 0.5 to 1, where 0.5 indicates no discrimination, and 1 represents perfect discrimination of the model. Results were interpreted with the ranking by Lemeshow et al. in mind [52]. However, to adapt the ranking of AU-ROC levels to a social epidemiological context, modified cut-offs and labels were used according to those described by Axelsson Fisk et al. [38]. Here, the discriminatory accuracy was thus considered ‘absent or very small’ if AU-ROC =0.5–0.6, ‘moderate’ if AU-ROC > 0.6–≤0.7, ‘large’ if AU-ROC > 0.7–≤0.8, and ‘very large’ if AU-ROC > 0.8. The AU-ROC was calculated separately for each of the outcome variables according to three models. The first model included only the covariates, while the second model included the covariates and education and gender as binary variables. The third model comprised the covariates and the cross-classified intersectional variables. A descriptive estimate of the change in the ROC area with Model 1 as reference was also calculated, expressed as Δ AU-ROC.

## Results

### Descriptive data analysis

The descriptive statistics for the population studied are displayed in Table 1. High educated women made up 22.5% (*n* = 4908) of the final population retained for analysis. An additional 31.3% (*n* = 6849) were low educated women, and high educated and low educated men accounted for 14.4% (*n* = 3147) and 31.8% (*n* = 6949) respectively.

**Fig. 1:**
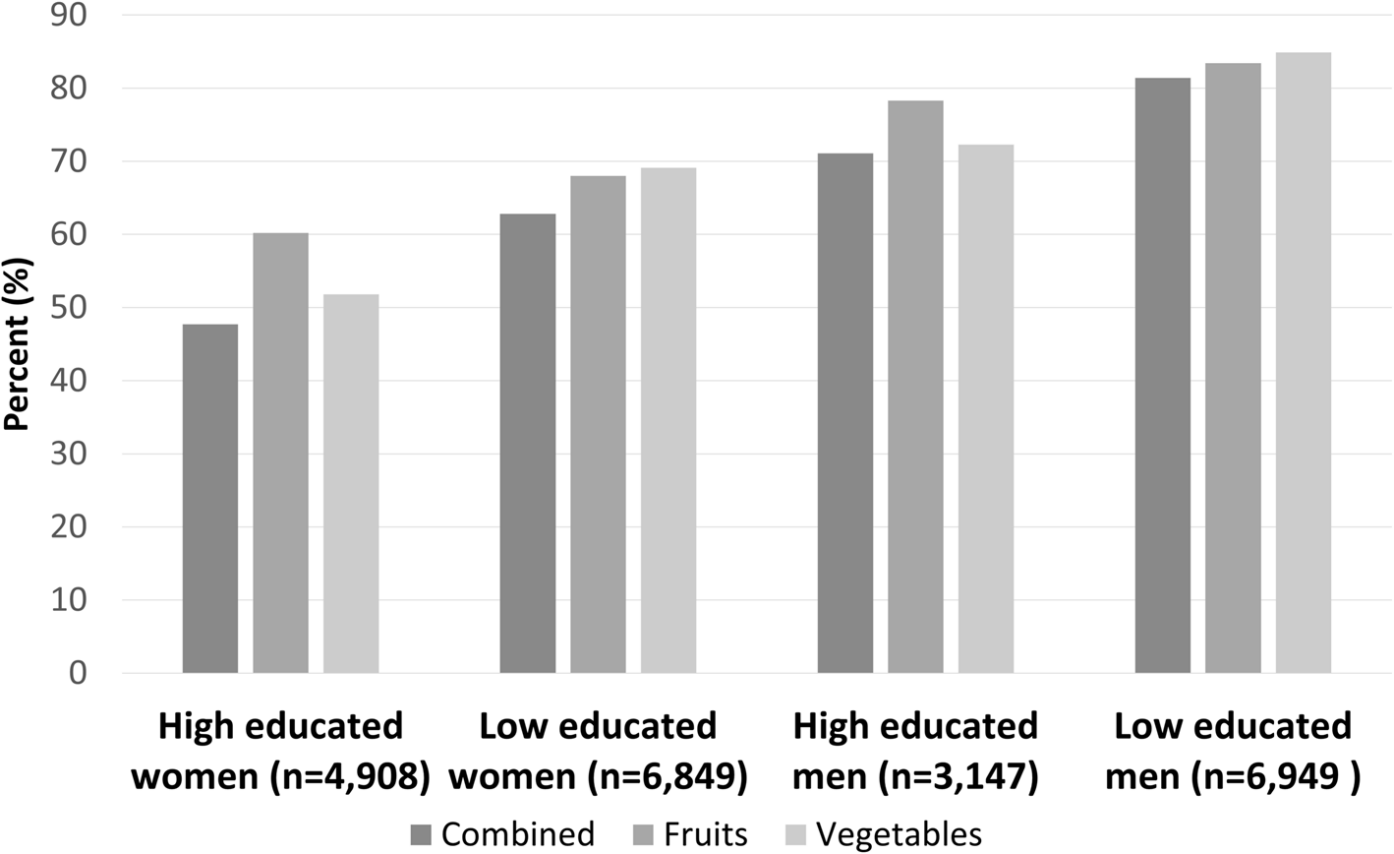
Prevalence of inadequate intake of fruits and vegetables combined, fruits, and vegetables, by intersectional group by education and gender (*N* = 21,853)

The prevalence of inadequate intake of fruits and vegetables combined, fruits, and vegetables was highest among low educated men (81.4, 83.4, and 84.9% respectively) and lowest among high educated women (47.7, 60.2, and 51.8% respectively) (Fig. 1).

Overall, 58.3% (*n* = 12,741) reported an inadequate intake of both fruits and vegetables, while 15% (*n* = 3270) reported an adequate intake of both. The remaining 26.7% (*n* = 5842) reported an adequate intake of one but not the other (cross-tabulation available upon request).

### Intersectional disparities in fruit and vegetable intake

**Fig. 2:**
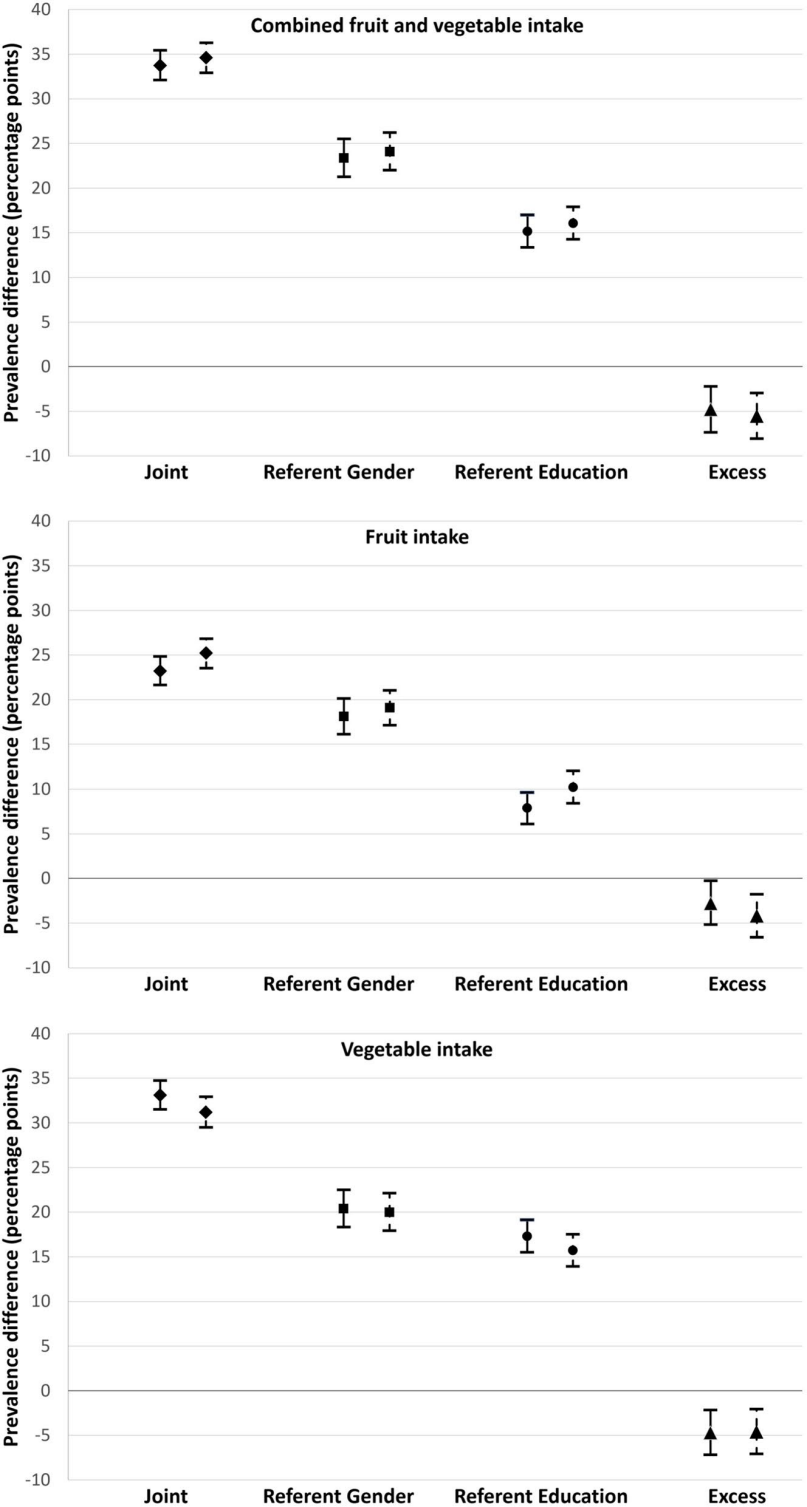
Disparities in consumption between intersectional groups by education and gender in crude models (solid line) and models adjusted for age, civil status and region of residence (dashed line). Estimates are prevalence differences expressed in percentage points, with 95% confidence intervals

The crude and adjusted analysis yielded similar results (Table 2) (Fig. 2), indicating that the covariates did not play a strong confounding role. The direction and level of significance of the results were similar, although not identical, between analysis for each outcome variable and each disparity studied.

**Table 2:** Crude and adjusted intersectional disparities in consumption of fruits and vegetables combined, fruits, and vegetables

Disparity	Prevalence difference in inadequate intake ^a^											
	Fruits and vegetables (combined)				Fruits				Vegetables			
	Crude^b^		Adjusted^c^		Crude^b^		Adjusted^c^		Crude^b^		Adjusted^c^	
	pp. [CI]	*p*-value	pp. [CI]	*p*-value	pp. [CI]	*p*-value	pp. [CI]	*p*-value	pp. [CI]	*p*-value	pp. [CI]	*p*-value
Joint^d^	33.7 [32.1 to 35.4]	< 0.001	34.6 [32.9 to 36.3]	< 0.001	23.2 [21.6 to 24.8]	< 0.001	25.2 [23.5 to 26.8]	< 0.001	33.1 [31.5 to 34.7]	< 0.001	31.2 [29.5 to 32.9]	< 0.001
Referent gender	23.4 [21.3 to 25.5]	< 0.001	24.1 [22.0 to 26.2]	< 0.001	18.1 [16.1 to 20.1]	< 0.001	19.1 [17.1 to 21]	< 0.001	20.4 [18.3 to 22.5]	< 0.001	20 [17.9 to 22.1]	< 0.001
% of joint disparity	69		70		78		76		62		64	
Referent education	15.2 [13.4 to 17.0]	< 0.001	16.1 [14.2 to 17.9]	< 0.001	7.9 [6.1 to 9.6]	< 0.001	10.2 [8.4 to 12]	< 0.001	17.3 [15.5 to 19.1]	< 0.001	15.7 [13.9 to 17.5]	< 0.001
% of joint disparity	45		46		34		40		52		50	
Excess	−4.8 [-7.4 to −2.2]	< 0.001	−5.5 [-8.1 to −3.0]	< 0.001	− 2.8 [-5.2 to −0.3]	0.026	− 4.2 [-6.6 to −1.8]	0.001	−4.7 [-7.2 to − 2.2]	< 0.001	−4.6 [-7.1 to − 2.1]	< 0.001
% of joint disparity	−14		−16		−12		−17		−14		− 15	

^a^ Estimates are percentage points (pp.) and 95% confidence intervals [CI] unless otherwise specified

^b^ Crude models including the cross-classified intersectional variable only

^c^ Adjusted models including the cross-classified intersectional variable adjusted for age, civil status, and region of residence

^d^ Joint disparity = Referent disparity for gender + Referent disparity for education + Excess intersectional disparity

#### Combined fruit and vegetable intake

In the adjusted analysis, the joint disparity in combined fruit and vegetable intake estimated a 34.6 percentage points (pp.) (95% CI: 32.9 to 36.3) higher prevalence of inadequate intake of fruits and vegetables among low educated men than among high educated women. The decomposition of the joint disparity showed that the referent disparity for gender made a greater contribution to the joint disparity (24.1 pp.; 95% CI: 22.0 to 26.2) than the referent disparity for education (16.1 pp.; 95% CI: 14.2 to 17.9). Furthermore, the excess intersectional disparity indicated a marked lower prevalence (− 5.5 pp.; 95% CI: − 8.1 to − 3.0) of inadequate combined intake of fruit and vegetable in low educated men than what would be expected from the addition of the two referent disparities.

#### Fruit intake

The results for fruit intake took a similar direction, although the adjusted joint disparity was much smaller than for combined intake (25.2 pp.; 95% CI: 23.5 to 26.8). Again, as for combined intake of fruits and vegetables, the referent disparity for gender (19.1 pp.; 95% CI: 17.1 to 21) was greater than the referent disparity for education (10.2 pp.; 95% CI: 8.4 to 12). The excess intersectional disparity (− 4.2 pp.; 95% CI: − 6.6 to − 1.8) was also akin to the one observed for combined intake, indicating that the prevalence of inadequate fruit intake in low educated men was lower than what could be expected from the addition of the referent disparities for gender and education.

#### Vegetable intake

Similarly, the joint disparity in vegetable intake in the adjusted analysis (31.2 pp.; 95% CI: 29.5 to 32.9) indicated that inadequate intake of vegetables was significantly more prevalent in low educated men than in high educated women. However, the joint disparity for vegetable intake was much higher than for fruit intake. The referent disparity for gender (20 pp.; 95% CI: 17.9 to 22.1) and the referent disparity for education (15.7 pp.; 95% CI: 13.9 to 17.5) also followed a similar pattern to the other outcomes, with the referent disparity for gender being the largest of the two. Also in this case, the excess intersectional disparity left after subtracting the two referent disparities (− 4.6 pp.; 95% CI: − 7.1 to − 2.1) suggested that inadequate intake of vegetables among low educated men was less prevalent than expected from the addition of the two referent disparities.

### Discriminatory accuracy

The AU-ROC increased substantially for all three outcomes from Model 1 to Model 2 or from Model 1 to Model 3 (Table 3). For combined fruit and vegetable intake, the discriminatory accuracy as measured by the AU-ROC was 0.54 (‘absent or very small’) when including only the demographic covariates, and 0.67 (‘moderate’) when adding independent variables in Model 2. It remained unchanged when taking the cross-classified intersectional positions into account in Model 3.

**Table 3:** Discriminatory accuracy of variables estimated by the Area Under the Receiver Operating Characteristic curve (AU-ROC)

Outcome variables	AU-ROC [95% Conf. Interval]		
	Model 1^a^	Model 2^b^	Model 3^c^
Combined intake	0.54 [0.53 to 0.54]	0.67 [0.66 to 0.68]	0.67 [0.66 to 0.68]
Δ AU-ROC	Reference	0.1308	0.1309
Fruit intake	0.56 [0.55 to 0.57]	0.65 [0.64 to 0.66]	0.65 [0.65 to 0.66]
Δ AU-ROC	Reference	0.0898	0.0902
Vegetable intake	0.58 [0.57 to 0.59]	0.68 [0.67 to 0.68]	0.68 [0.67 to 0.68]
Δ AU-ROC	Reference	0.0962	0.0962

^a^ Model 1: age, civil status, and region of residence

^b^ Model 2: model 1 + education + gender

^c^ Model 3: model 1 + cross-classified intersectional groups

## Discussion

This study aimed to estimate disparities in fruit and vegetable intake between groups at the intersection of educational level and gender in northern Sweden, and to assess the discriminatory accuracy of the intersectional groups. Substantial differences between intersectional groups were found in the prevalence of inadequate intake of fruits and vegetables combined, and separately. Gender made a greater contribution overall to the disparities than education did, and the excess intersectional disparities were negative and similar across outcomes. In addition, the magnitude of the joint disparities varied by outcome. The discriminatory accuracy of the covariates and gender and education was moderate and suggested an importance of these two inequality dimensions, but their cross-classification did not contribute to any additional discriminatory power.

The finding that the joint disparity for fruit intake was lower compared to the other outcomes can be explained by a higher prevalence of inadequate intake among high educated women compared to the two other outcomes (60.2% for fruit intake, vs 47.7, and 51.8% for the other two), whereas it was similar across outcomes for low educated men (83.4% for fruit intake, vs 81.4 and 84.9% for the other two).

We also found that the referent disparity for gender consistently explained a large portion of the joint disparity. This is in line with previous research indicating that women have in general a higher fruit and vegetable consumption than men [6, 19, 20], and healthier behaviors overall [15–18]. Although to a lesser extent, the referent disparity for education also explained a substantial fraction of the joint disparities. This is consistent with the literature on educational inequalities in fruit and vegetable consumption [6–10, 12, 13], and other types of health behaviors [11, 16, 53]. Several potential mechanisms have been proposed to explain this relationship between education level and health behaviors [14].

The finding that the excess intersectional disparity was similar across outcomes illustrates the partial overlap that exists between fruit and vegetable intake. The contribution of the excess intersectional disparities to the joint disparities observed here points to the potential usefulness of intersectional approaches in epidemiology. The intersection of multiple disadvantages for fruit and vegetable intake appeared to be associated with a unique pattern of consumption not entirely explained by the addition of gender and education: the intersection of male gender and low education appeared to be unexpectedly protective against unhealthy dietary patterns, in comparison to what would be expected from the addition of the two referent disparities.

As pointed out by Jackson et al. [33], it should also be noted that the existence and magnitude of the joint disparity should not be dismissed if the excess intersectional disparity is non-existent or negative. Even if a group does not display any excess intersectional disparity, it can still experience the sum of the two referent disparities. In this study, the joint disparity in consumption of fruits, vegetables, and fruits and vegetables between high educated women and low educated men was still great in magnitude; inadequate intake of fruits and vegetables combined was 34.6 pp. more prevalent for low educated men than for high educated women.

The discriminatory accuracy of the intersectional groups for identifying inadequate intake of fruits and vegetables as measured by the AU-ROC was close to - but below - 0.7 and could therefore be considered moderate. Most importantly, the lack of additional discriminatory power of the cross-classification found in this study is recurrent in empirical studies using intersectional approaches to study inequalities in Sweden [34, 38, 39]. Those two findings would be commonly interpreted as evidence in favor of universal rather than targeted intervention in the context of proportionate universalism. For the present study, this would suggest that the targeting of specific intersectional groups is not suitable as the sole guiding principle for health promotion planning. However, while this interpretation is straightforward when the goal is general health promotion in the population, e.g., increase fruit and vegetable intake, it is not as evident when the goal is promotion of equity in health, e.g., reducing inequalities in fruit and vegetable intake.

Moreover, there is no clear-cut threshold that would label a discrimination as high enough for intersectional approaches in epidemiology. Indeed, examinations of both novel and traditional risk factors for coronary heart disease have yielded similarly disappointing results when it comes to discriminatory accuracy [54], despite many of them being established in clinical practice. While discriminatory accuracy represents a necessary complement to means-centric examinations of health inequalities, and to traditional epidemiology more generally, a cautious interpretation of its implications specifically in the context of equity promotion is therefore warranted.

### Further research and public health relevance

To our knowledge, this Swedish study is the first of its kind investigating disparities in fruit and vegetable consumption across intersectional groups defined by gender and education. The intersectional approach was helpful to shed light on the complex inequality patterns of inadequate intake of fruits and vegetables. In addition, as the estimated disparities are additive rather than relative measure of inequality, they can be used to determine the absolute benefits in population health if a disparity were to be eliminated [33].

The findings from this study will hopefully help to give a more nuanced understanding of the structural patterning of disparities in diet, and thereby improve the effectiveness of equity-promoting public health interventions and policies. Although the moderate discriminatory accuracy in this study could suggest that universal interventions and policies might be more effective for improving diet quality in Sweden, there is a risk that they would mostly benefit those already advantaged, and could therefore contribute to widening inequalities in health [55]. A combination of universal and targeted measures to improve diet quality could be a way forward, in line with proportionate universalist principles [56]. This study could then contribute to giving guidance as to where are the largest disparities, and what are the most relevant groups to prioritize.

Future research on diet quality could consider operationalizing groups at the intersection of gender, education, and other inequality dimensions such as age, sexual orientation, migration and ethnicity, or geographical area of residence. This could help identify intersectional groups with higher discriminatory accuracy and facilitate targeted interventions to improve health equity. In addition, qualitative or mixed methods could be considered, as they could shed light on the perceptions and motivations of individuals located at specific intersections of social positions and identities that may explain the disparities observed in this study.

### Strengths and limitations

Since random sampling procedures were used to draw a sample from the general population, selection bias is limited to some extent. However, the response rate varied between 42.4% for Västerbotten [46] and 45.8% for Jämtland/Härjedalen [44]. Since most people contacted to answer the survey did not answer it, it is unknown whether the final sample is truly representative of the target population.

The year when the Health on Equal Terms survey used in this study was conducted, 43% of the population aged 25–64 had some form of post-secondary education [57]. In our study, 37% are in that same category (Table 1), which is slightly lower but can be explained by the presence of survey participants older than 64 years old and by the increase in education level in the last decades [57].

It is not known to which degree the results can be generalized outside of Sweden. Indeed, the implications of the intersection of gender and education can be expected to differ between contexts, depending on the specific configuration of the gender (in)equality structure, of the educational system, and of equity-promoting policies. In addition, the results cannot readily be generalized to other countries and settings without consideration of the different patterns of health and nutrition [58–60].

The use of gender as one of the two variables for the operationalization of the intersectional groups can be questioned. Since this study was interested in investigating social inequalities in health behaviors, and not potential biological inequalities in health, gender seemed more appropriate as designating a social construct rather than a biological construct. However, what is measured by available register data is biological sex recorded at birth, and thus might not always represent gender identity accurately, especially since gender identities cannot be fully captured by a binary variable. The adequateness of using one term or the other in epidemiology has been discussed elsewhere [61].

In addition, dichotomizing educational attainment may have led to loss of information, since individuals with different educational characteristics were categorized into the same group. For example, individuals who did not complete high school were grouped together with individuals who did. The patterns of fruit and vegetable consumption might differ between those two groups, but this cannot be inferred from the present study.

The three outcome variables in this study were dichotomized as well, also potentially leading to loss of information. However, we dichotomized the outcome for combined intake of fruit and vegetable based on recommendations from the Public Health Agency of Sweden [43]. In addition, because one serving reported in the survey is usually estimated to be close to 100 g [43], fruit and vegetable intake resembled amounts recommended in Sweden and worldwide. Defining adequate intake as “twice a day or more” for fruit and vegetable (each) theoretically yields 400 g or more in total. This is close to the 500 g recommended in Sweden [5] and to the 400 g or 5 servings recommended by the World Health Organization [3]. Moreover, the three outcomes showed a similar prevalence of inadequate intake (Table 1). The choice of dichotomization for the outcomes is therefore intended to correspond to policy recommendations.

However, although it was assumed that the frequency of fruit and vegetable intake reported was, on average, proportional to the amount consumed, we could not assess whether this was in fact the case. Reporting bias could be a concern and over-reporting may have, for example, occurred more in those with higher education since they might be more conscious of the existing recommendations for fruit and vegetable intake. Over-reporting may also have occurred due to social desirability bias [62]. Under-reporting may have occurred among those with relatively lower education due to potentially weaker health and nutrition knowledge. Misreporting could also have occurred for other reasons, such as having a high body mass index or obesity [63], or cognitive decline in older adults in the sample, although those older adults may have not answered the survey to start with. This could therefore contribute to selection bias rather than information bias. In contrast, the exposure variables and covariates came from high-quality register data and therefore were not affected by self-report bias.

Since this study is cross-sectional in design, it cannot be used to determine causality, even though reverse causality is unlikely. In addition, despite adjusting for additional covariates, it is not impossible that some residual confounding may subsist. However, the associations found between intersectional group and prevalence of inadequate fruit and vegetable intake are not easily affected by confounders. Indeed, most third variables such as income or occupation are likely to be determined in part by gender and education, and if included would risk over-adjustment and result in under-estimation of the disparities.

Finally, the method described by Jackson et al. [33] allows to quantitatively investigate disparities at the intersection of multiple social positions and identities, in a way that corresponds to the different possibilities for an intersectional group to experience disadvantage [24]. However, this approach limits the number of social positions that can be considered simultaneously [33]. In addition, it measures additive interactions, whereas the AU-ROC is based on multiplicative interactions. This may partly explain why measures of discriminatory accuracy did not identify any intersectional interaction from Model 2 to Model 3, whereas substantial excess intersectional disparities were estimated for the three outcomes.

## Conclusions

This study found substantial differences in the prevalence of inadequate intake of fruits and vegetables, favoring high educated women, and disfavoring low educated men. Gender seemed to have a greater impact than education in explaining the disparity. The moderate discriminatory accuracy seen in this study suggests that interventions and policies aiming to reduce diet inequalities should not solely be targeted at certain groups, but also be universal. The findings should be relevant for public health policies and interventions whose goals are to reduce inequalities in health by providing a more accurate picture of the social patterning of consumption of fruits and vegetables.

## Data Availability

The data that support the findings of this study are available from the respective Region of Norrbotten (www.norrbotten.se), Västerbotten (www.regionvasterbotten.se), Jämtland/Härjedalen (www.regionjh.se) and Västernorrland (www.rvn.se), but restrictions apply to the availability of these data as regulated by the Public Access to Information and Secrecy Act (2009:400) and European General Data Protection Regulation (2016/679, GDPR). The researchers of this study were, after mandatory ethical approval by the Swedish Ethical Review Authority, granted access to the data for research purposes according to the data protection policy of Umeå University. Data are however available from the authors upon reasonable request and with permission of the Regions of Västerbotten, Norrbotten, Jämtland/Härjedalen and Västerbotten and of Umeå University.

## Abbreviations

AU-ROC: Area Under the Receiver Operating Characteristic curve
CI: Confidence Interval
(M)AIHDA: (Multilevel) Analysis of Individual Heterogeneity and Discriminatory Accuracy
ROC: Receiver Operating Characteristic
Δ AU-ROC: change in the ROC Area with Model 1 as reference
